# Multi-Directional Scene Text Detection Based on Improved YOLOv3

**DOI:** 10.3390/s21144870

**Published:** 2021-07-16

**Authors:** Liyun Xiao, Peng Zhou, Ke Xu, Xiaofang Zhao

**Affiliations:** 1 Institute of Engineering Technology, University of Science and Technology Beijing, Beijing 100083, China; S20191258@xs.ustb.edu.cn (L.X.); s20191259@xs.ustb.edu.cn (X.Z.); 2 Beijing Key Laboratory of Knowledge Engineering for Materials Science, Institute of Artificial Intelligence, University of Science and Technology Beijing, Beijing 100083, China; 3 Collaborative Innovation Center of Steel Technology, University of Science and Technology Beijing, Beijing 100083, China; xuke@ustb.edu.cn

**Keywords:** multi-directional text detection, natural scenes, YOLOv3, CIOU

## Abstract

To address the problem of low detection rate caused by the close alignment and multi-directional position of text words in practical application and the need to improve the detection speed of the algorithm, this paper proposes a multi-directional text detection algorithm based on improved YOLOv3, and applies it to natural text detection. To detect text in multiple directions, this paper introduces a method of box definition based on sliding vertices. Then, a new rotating box loss function MD-Closs based on CIOU is proposed to improve the detection accuracy. In addition, a step-by-step NMS method is used to further reduce the amount of calculation. Experimental results show that on the ICDAR 2015 data set, the accuracy rate is 86.2%, the recall rate is 81.9%, and the timeliness is 21.3 fps, which shows that the proposed algorithm has a good detection effect on text detection in natural scenes.

## 1. Introduction

Text is the carrier of information exchange and widely exists in natural scenes [1]. How to accurately locate the text position in complex natural scenes is the basis and premise of text recognition, and it is the key step to obtain information in the scene. With the rapid development of deep learning and computer vision, related technologies have been widely used in many fields. Object detection is an important part of image understanding. Its task is to find all the objects of interest in the image, and to determine their position and size, which is one of the core problems in the field of machine vision [2,3]. Scene text detection is a type of object detection [4], and the positioning box category is divided into two types: text area and background. The general target detection method is to locate the object by generating a horizontal rectangular box, such as the classic detection algorithm faster-rcnn [5], yolo series [6,7,8], SSD [9], and wait. When they are applied to natural scene text detection, facing characters and words are closely arranged and the positions are in multiple directions, the generated horizontal positioning box will inevitably cover multiple words, resulting in inaccurate positioning and poor applicability of the algorithm. Therefore, it is necessary to adopt a multi-directional target detection algorithm, that is, to locate the scene text by generating an angled rectangular box or an arbitrary quadrilateral box.

In recent years, scholars have achieved certain results in the research of multi-directional target detection algorithms and scene text detection algorithms. Zhi et al. [10] proposed the connectionist text proposal network, introduced LSTM into the network to play the memory function of LSTM, and extracted this mutual relationship feature according to the anchor sequence before and after, and finally used the text line construction method to connect the anchors to obtain text lines, but this method was only suitable for slightly slanted text detection. Shi et al. [11] proposed SegLink, which detects text in segments, and then connects them according to rules, so that text of any length can be detected. Jian et al. [12] proposed a novel framework, namely TextFuseNet, to exploit the use of richer features fused for text detection. Linjie et al. [13] proposed convolutional character networks, referred as CharNet to achieve scene text detection and recognition at the same time. Yuliang et al. [14] noticed the significance of consistent labeling and proposed a new method (OBD) to ensure a stable training process. Ma et al. [15] used the form of a rotating rectangular box for detection. Based on Faster RCNN, they proposed a rotating candidate area network RRPN to generate angled anchors for prediction. However, due to the five-parameter box definition method, there will be angle boundary problems. In order to adapt to the task of detecting multi-angle texts, Liao et al. [16] changed the SSD algorithm and used two methods to return to the multi-angle text box based on the default box. When the regression method of predicting quadrilateral vertices is adopted, there will be a vertex sorting problem. The Gliding_vertex algorithm was proposed by Xu et al. [17]. It has outstanding performance in the field of rotating target detection under complex backgrounds. It is based on the high-precision second-order detection algorithm Faster RCNN, and detects rotating targets based on the existing box definition methods. For the problem, the definition method of the rotating box of the sliding vertex is proposed, which avoids the angle boundary problem and the vertex ordering problem, and thus is well adapted to various application scenarios of multi-directional target detection. The YOLO series has a fast recognition speed and is widely used in target detection. In particular, the YOLOv3 algorithm has high real-time performance while still maintaining high detection accuracy. To this end, this article will improve the YOLOv3 algorithm, take advantage of its high efficiency and accuracy, increase the ability of rotating target detection, so that the algorithm can be applied to multi-directional text detection in natural scenes.

## 2. The Analysis of YOLOv3 Algorithm

**Figure 1:**
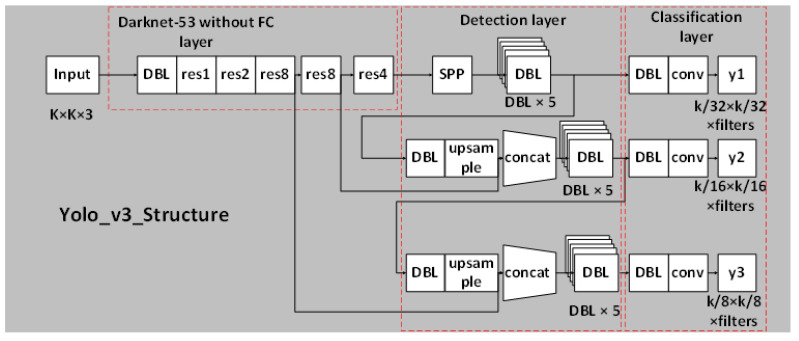
YOLOv3 network structure diagram.

In order to satisfy the detection of scene text, this paper adopts the YOLOv3 network in the YOLO series. At present, in the mainstream target detection network, the YOLO network directly performs regression detection on the target in the image, with no RPN network and no preset boxes, so their detection speeds are faster than other networks. YOLOv3 is an excellent representative among the YOLO series. It achieves a clever balance of detection speed, accuracy, and model deployment difficulty, and has good performance. It is currently one of the most popular and widely used target detection algorithms. The YOLOv3 network model is shown in Figure 1. Its basic architecture includes three main parts: the feature extraction network layer (Darknet-53), the detection layer and the classification layer.

In Figure 1, DBL is the basic component of Yolov3, which is composed of Convolutional layer [18], BN layer, and Leaky ReLU layer.

Feature extraction network layer: this is a feature extraction algorithm named DarkNet-53. It draws on the practice of the residual network (ResNet) to establish shortcut links between some layers. The short-cut link layer connection can solve the problem that the model is difficult to optimize with the gradual deepening of the network, reduce the risk of gradient explosion, strengthen the learning ability of the network, and use more shallow image feature information. For the next step of the YOLOv3 network, the feature extraction network part will output three feature maps of different sizes as the input of the next network module, the sizes of which are respectively 8 times, 16 times and 32 times smaller than the original image size.

Detection layer: convolutional layers with convolution kernel sizes of 1 × 1 and 3 × 3 appear alternately, and the convolution kernel size of the last layer must be 1 × 1. The feature map with the smallest input scale is only processed in the detection layer. The feature maps of the other two scales are first spliced with the lower dimensional maps, and then input to the detection layer.

Classification layer: the fusion feature containing multi-scale feature information generated by the detection layer is used as input, and the final feature output of the model is generated through the convolutional layer with the convolution kernel size of 3 × 3 and 1 × 1. The number of channels (filters) in the last layer is as follows:

(1) $$Filters=3*\left({\left(x,y,w,h\right)}_{box}+confidence+class\right),$$

where: 3 represents 3 prediction scales, ${\left(x,y,w,h\right)}_{box}$ is the normalized center coordinates (x,y,w,h) of the network’s final output detection target category, so it occupies 4 channels. confidence occupies the number of channels, and class represents the number of detection target categories. In this study, the boxes position coordinates are $\left(x,y,w,h,\varepsilon_{1},\varepsilon_{2},\varepsilon_{3},\varepsilon_{4},r\right)$, and there are two detection categories, so filters = 36.

## 3. The Algorithm Design of This Paper

### 3.1. Sliding Vertex Box Definition Method

Based on YOLOv3, this article uses a sliding vertex box definition method that is different from the general rotation box definition method to better adapt to multi-directional text detection.

**Figure 2:**
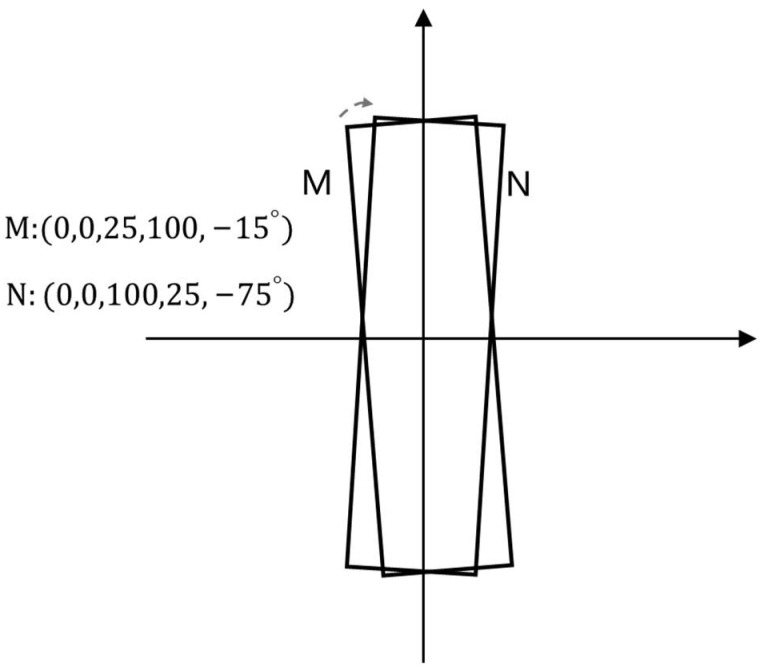
Auxiliary illustration of five-parameter method defects.

The frame definition method is the basic item of the multi-directional target detection algorithm that is different from the horizontal frame target detection. Generally, the horizontal frame is defined as (x,y,w,h), and the rotation frame is defined in two ways: five-parameter method (x,,w,h,θ) and eight-parameter method $\left(x_{1},y_{1},x_{2},y_{2},x_{3},y_{3},x_{4},y_{4}\right)$. The two methods have the following problems:

(1) The angle θ of the five-parameter method refers to the acute angle formed by the frame and the *x*-axis, and this side of the frame is denoted as w and the other side is denoted as h. The range of the angle is $\left(-90^{{}^{\circ}},0\right)$. Taking Figure 2 as an example, M is the candidate frame and N is the real frame. The original optimal angle regression route is shown by the dashed arrow in Figure 2, that is, only the angle regression is required. However, according to the definition of the five-parameter method, the coordinates of the two frames are shown in Figure 2, which leads to the need for regression adjustment for the parts w,h,θ, that is, the regression route is unreasonable.
(2) The eight-parameter method needs to sort the vertices of the prediction box, which increases the amount of calculation. The prediction frame coordinates obtained through the network forward propagation are not arranged in order according to the coordinate position. The four coordinate points of each prediction frame need to be sorted and compared to obtain the first coordinate point (usually put the upper left corner point in the first place). This leads to an increase in the amount of calculation.

In order to detect the rotating target, the multi-direction scene text detection algorithm proposed in this paper applies the sliding vertex rotation frame definition method to the yolo layer of the YOLOv3 detection head. The output of the network is an arbitrary quadrilateral prediction box, represented by parameters $\left(x,y,w,h,\varepsilon_{1},\varepsilon_{2},\varepsilon_{3},\varepsilon_{4},r,confidence,cls_{0},cls_{1}\right)$, where $cls_{0},cls_{1}$ represent the probability that the prediction box is background and text, confidence represents the position confidence of the prediction box, and other parameters are defined below.

**Figure 3:**
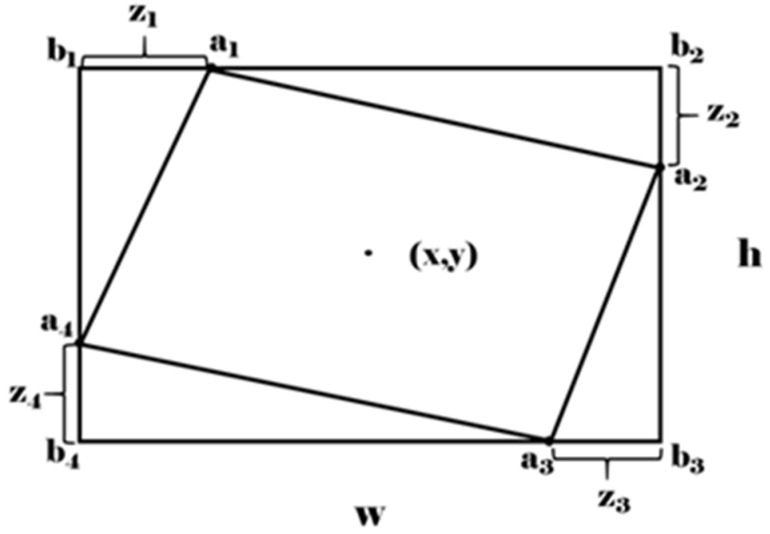
Schematic diagram of the sliding vertex box definition method.

The definition of the sliding vertex rotation box is shown in Figure 3, where the horizontal box parameters are (x,y,w,h), and the parameters $\varepsilon_{i},i\in \left\{1,2,3,4\right\}$ are obtained by Formulas (2) and (3):

(2) $$\varepsilon_{\left\{1,3\right\}}=z_{\left\{1,3\right\}}/w,$$

(3) $$\varepsilon_{\left\{2,4\right\}}=z_{\left\{2,4\right\}}/h,$$

In the above formula, $z_{i}=\left|\left|a_{i}-b_{i}\right|\right|$ is the offset value of the vertex $a_{i}$ of the rotating box A relative to the vertex $b_{i}$ of the horizontal box B. In addition, set the rotation factor r=|A|/|B| to indicate the degree of rotation of the rotating box, when r is greater than the set threshold (threshold of r is 0.85 in this article), the horizontal box parameter is used to represent this prediction box.

At present, the position label format provided by most multidirectional data sets is the four-vertex coordinates of the box $\left(x_{1},y_{1},x_{2},y_{2},x_{3},y_{3},x_{4},y_{4}\right)$. Therefore, before model training, it is necessary to increase the ground truth preparation stage, which is converting $\left(x_{1},y_{1},x_{2},y_{2},x_{3},y_{3},x_{4},y_{4}\right)$ to $\;\left(x,y,w,h,\varepsilon_{1},\varepsilon_{2},\varepsilon_{3},\varepsilon_{4},r\right)$.

This frame definition method does not involve angle parameters, so it avoids the angle boundary problem of the five-parameter method. In addition, the loss during network training is directly calculated from the true deviation value and the predicted deviation value, and the amount of calculation is lower than the eight-parameter method, so that the entire algorithm has a good foundation for multi-directional text detection.

### 3.2. Location Loss Function

**Figure 4:**
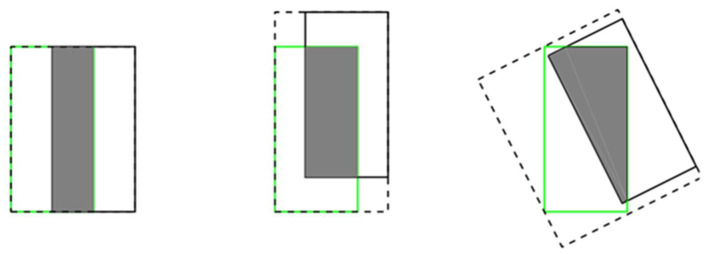
Three different crossover methods correspond to the same IOU value, value = 0.33.

In the updated YOLOv3 algorithm code, the position loss uses IOU loss for regression, but it is only suitable for horizontal rectangular boxes and cannot adapt to the rotating target detection algorithm, and IOU loss has the following two defects:

(1) If the two targets do not overlap, IOU will be 0. At this time, regardless of the distance between the two boxes, the IOU cannot be reflected. If IOU is used as the loss function, the gradient is 0 and cannot be optimized;
(2) IOU cannot distinguish between different alignments between two objects. More precisely, the IOU of two overlapping objects with the same level of intersection in different directions will be exactly the same, as shown in Figure 4.

The design of CIOU loss can solve the problems of IOU, and consider the aspect ratio of the predicted frame to fit the aspect ratio of the target frame, which can better reflect the position difference. The expression of CIOU loss is shown below (4)–(6), where α and ν are the impact factors, where α is the parameter used for trade-off, and ν is the parameter used to measure the consistency of the aspect ratio.

(4) $$L_{CIOU}=1-IOU+\frac{\rho^{2}\left(b,b^{gt}\right)}{c^{2}}+\alpha \nu ,$$

(5) $$\alpha =\frac{\nu }{\left(1-IOU\right)+\nu },$$

(6) $$\nu =\frac{4}{\pi^{2}}{\left(arctan\frac{w^{gt}}{h^{gt}}-arctan\frac{w}{h}\right)}^{2},$$

In this paper, combined with CIOU [19], a new position bounding box loss function MD-CLoss (multi-directional CIOU-based position loss function) is proposed to overcome the above two problems and applied in the second stage of the algorithm, expressed as follows:

(7) $$MD-CLoss=\lambda_{1}L_{h}+\lambda_{2}L_{\varepsilon }+\lambda_{3}L_{r},$$

(8) $$L_{h}=L_{\mathrm{CIOU}},$$

(9) $$L_{\varepsilon }=\sum_{i}^{4}smooth_{L_{1}}\left(\varepsilon_{i}-\varepsilon_{i}^{’}\right),$$

(10) $$L_{r}=smooth_{L_{1}}\left(r-r’\right),$$

The entire position loss function MD-CLoss is composed of horizontal box loss $L_{h}$, ε parameter loss $L_{\varepsilon }$, and rotation factor loss $L_{r}$. Refer to Section 3.2 for the definition of ε parameter and rotation factor r, $\lambda_{1},\lambda_{2},\lambda_{3}$ are hyperparameters used to balance MD−CLoss. ε parameter loss $L_{\varepsilon }$ and rotation factor loss $L_{r}$ make the loss value different when the relative position of the loss function to the quadrilateral frame to the horizontal frame is different.

The MD-Closs designed in this way can regress the rotating box, and effectively solve the problem of the same position loss corresponding to the different intersection of the candidate box and the real box, so that it can be adapted well to the multi-directional text box detection algorithm.

### 3.3. Step-by-Step Non-Maximum Suppression

**Figure 5:**
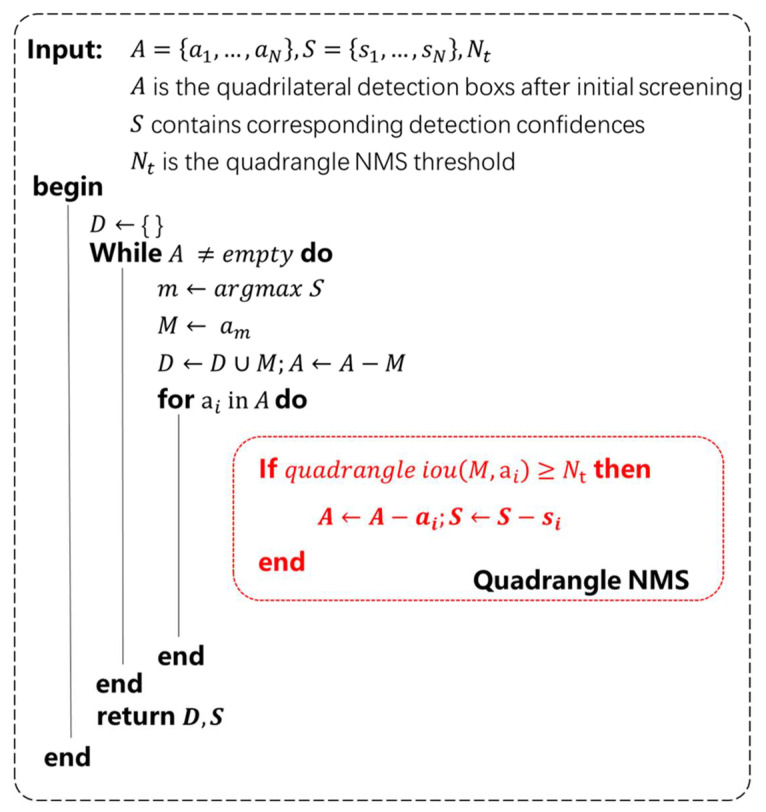
Algorithm step diagram of quadrangle NMS.

Non-maximum suppression has been proved very effective for the object detection task. Because of the particularity of the multi-direction scene text detection, rectangular NMS is limited to handle dense multi-oriented text. The position coordinates of the detection frame output in this paper are $\left(x,y,w,h,\varepsilon_{1},\varepsilon_{2},\varepsilon_{3},\varepsilon_{4},r\right)$, that is, the quadrilateral positioning frame is predicted on the basis of the horizontal rectangular frame. In view of the shortcomings of the rectangular NMS and considering the output of the algorithm in this paper, we divide the non-maximum suppression into two steps. Firstly, the prediction box is screened once using rectangular NMS with the horizontal rectangular parameters (x,y,w,h) as the index, and the threshold is 0.6; then, quadrangle NMS is performed on the deleted prediction box, and the threshold is 0.75. The quadrangle NMS steps are shown in Figure 5. In this way, NMS can be performed in steps to reduce the number of calculations of quadrilateral IOU and effectively increase the speed.

## 4. Experiment

### 4.1. Experimental Details

The experiment platform is as follows:

Processor: Intel Core i7-10700KF

Graphics card: NVIDIA GTX 1080Ti

Operating system: Ubuntu 16.04

Deep learning boxwork: Pytorch

System memory: 16G

In order to control the experimental variables, this article uses the same training method for the models (“Ours-I”, “Ours”) on the ICDAR2015 training data set; we use Adam as the network training optimizer. Adam optimizer is an optimization algorithm that finds the global best advantage. The algorithm introduces quadratic gradient correction. Compared with the basic SGD algorithm, Adam does not fall into local advantages easily and is faster. In the training process, the initial learning rate is set to 0.001, the learning rate adjustment strategy is steps, the maximum number of iterations is 65,000, and the learning rate is multiplied by 0.1 when the number of training iterations is 45,000 and 55,000, making the loss function further converge.

### 4.2. Data Set Introduction

**Figure 6:**
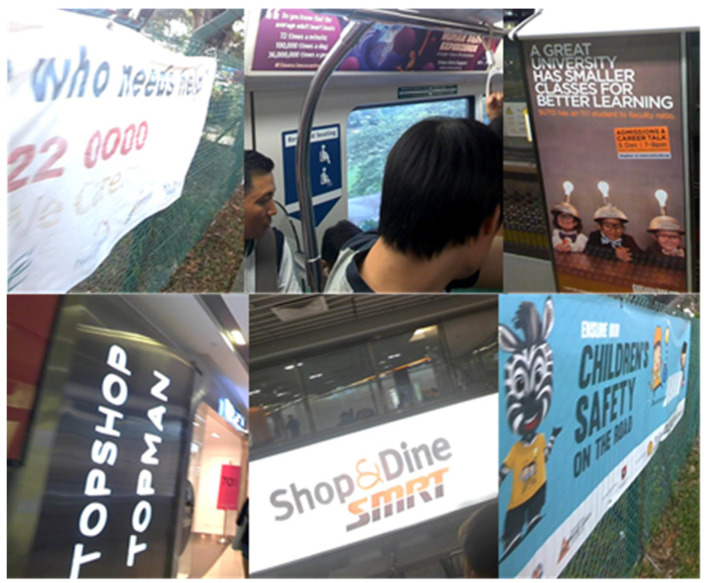
The background of the ICDAR 2015 data set is complex and changeable, the scale and direction of the text are arbitrary, and there is a phenomenon of tightly arranged positions.

The natural scene text localization data set used in this article is ICDAR2015. This data set is more popular in the field of scene text area detection, so the test results in this article have strong reference significance. ICDAR2015 has a total of 1000 training pictures and 500 test pictures. The text language is English. As shown in Figure 6, the background of the pictures is complex and changeable, the text scale and direction are arbitrary, and the text is closely arranged.

### 4.3. Algorithm Comparison

The evaluation indicators in this article: precision (*P*), recall (*R*) and average performance (*F*) are calculated by the following formulas:

(11) $$P=\frac{T_{C}}{T_{D}},$$

(12) $$R=\frac{T_{C}}{T_{G}},$$

(13) $$F=\frac{2(R\times P)}{R+P},$$

where: $T_{C}$ represents the total number of texts that can be correctly detected, $T_{D}$ represents the total number of texts detected by the algorithm, and $T_{G}$ represents the total number of detected texts contained in the test data set.

It can be seen from the formula that the accuracy rate P is the correct rate of detection of the text to be detected, and the recall rate  R relative to the accuracy rate *P* can reflect the missed detection rate of the text detection. When there are more missed texts in the data set, the corresponding *R* value is lower. When there are more misdetected texts in the data set, the corresponding *P* value is lower. The average performance *F* reflects the weighted and average between the accuracy rate *P* and the recall rate *R*. It can not only comprehensively reflect the results of the accuracy rate and the recall rate, but also represents the comprehensive performance of the algorithm. In the case of high *F*-average performance, it is proved that this algorithm has good detection results.

Use 1000 images of the ICDAR-2015 training set to train “Ours-I”, and “Ours” respectively. Subsequently, 300 images were randomly selected in the test set to test the model. The results obtained are compared with the mainstream algorithms SegLink, Hust_orientedText [20], SSTD [21], R2CNN [22], PixeLink [23] and the new models PAN [24] and DB (ResNet-50) [25]. In the selected 300 images, there are a total of 1984 text lines. The results are shown in Table 1. In the table, “Ours” refers to the final method proposed in this article, which is based on YOLOv3 using the sliding vertex box definition method, MD-CLoss and step-by-step NMS. Unlike “Ours”, “Ours-I” uses the smooth L1 function as the position loss, which is to verify the effectiveness of MD-CLoss.

Analysis of results: In Table 1, the “Ours” model is the model with the highest accuracy in the table, which is higher than the mainstream algorithms, with an accuracy value of 86.2%. Compared with PixeLink and DB (ResNet-50) in terms of recall rate and F value, although it is not dominant, it is much higher than the two models in terms of time performance. The specific comparison can be seen in Table 2. It can be shown that the proposed multi-directional scene text detection algorithm based on improved YOLOv3 has a good effect on the ICDAR2015 data set. At the same time, “Ours” has improved accuracy, recall rate, and F-measure than “Ours-I”, which shows the effectiveness of the loss function MD-Closs proposed in this paper.

### 4.4. Improvement Effect on Time Performance

The method of this article is not only accurate but also efficient. We have compared its runtime with the state-of-the-art methods on the ICDAR 2015 incidental text dataset. The results are shown in Table 2. The “Ours” method resizes the picture to 672 × 672 with a time performance of 21.3 fps. The comparison results are shown in Table 2.

In Table 2, Res represents the size of the input image, and Device displays the GPU version, which reflects the computing power of the device. It can be seen from the table that when the computing power of the equipment is not dominant, the speed of ‘ours’ is 21.3 fps as the best value in the table. The speed advantage is obvious. Analysis of the reason: the method in this paper is improved on the basis of YOLOv3, which has a very strong detection speed, and the step-by-step NMS method is used to further shorten the detection time in the NMS link with large computing power loss. Therefore, the algorithm in this paper has outstanding performance in terms of time performance.

### 4.5. Detection Effect

**Figure 7:**
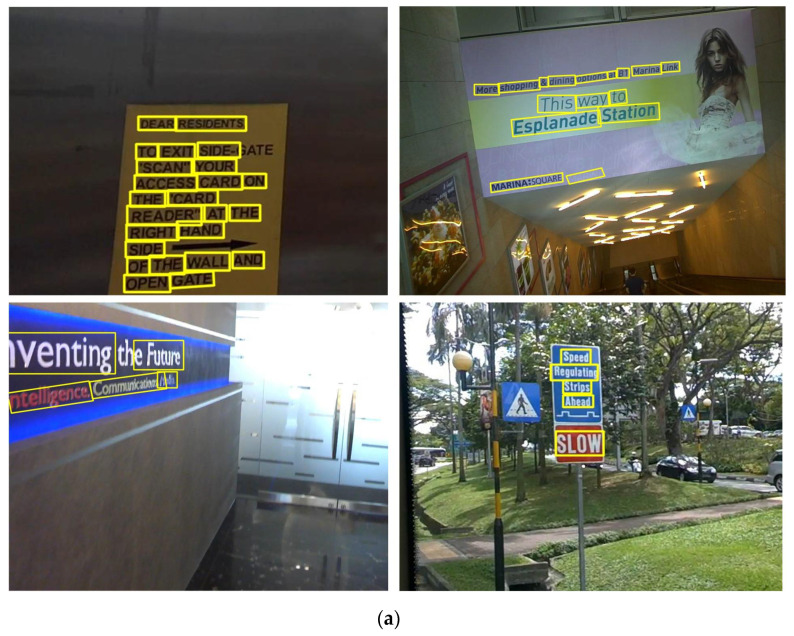

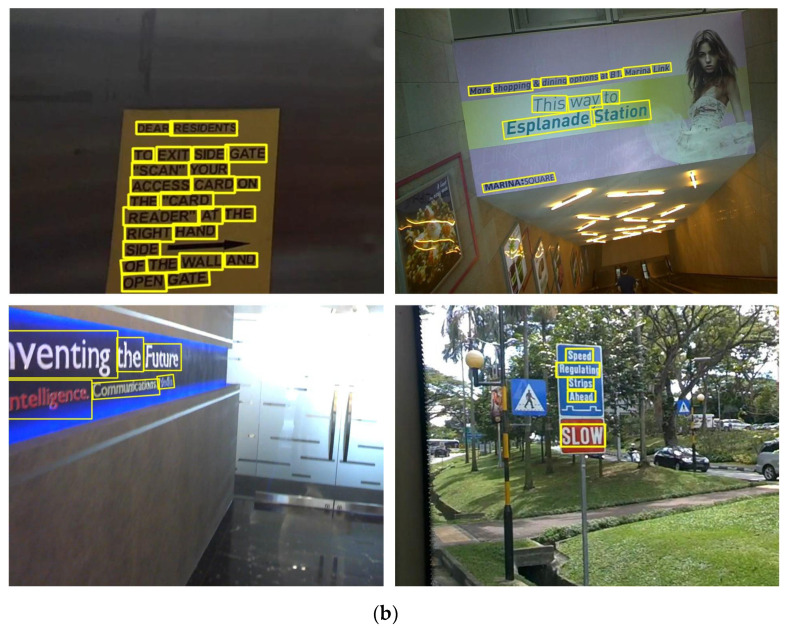
Comparison of detection results. Contains three sets of pictures, respectively, “Ours-I”, “Ours” detection effect chart. (**a**) The detection effect chart of “Ours-I”, (**b**) the detection effect chart of “Ours”.

Figure 7 shows the corresponding detection results of some test samples under the “Ours-I” and “Ours” models. The selected samples have the characteristics of dense arrangement, uneven light, non-right illumination pictures, and small scale. It can be seen from (a) that “Ours-I” can detect multi-directional text thanks to the sliding box definition method. However, it is easily affected by light causing missed detection and misdetection, especially for pictures that are not directly taken, but it has a good detection ability for small-scale text. (b) It reflects part of the detection effect of the final model “Ours” proposed in this paper. Compared with the “Ours-I” model, “Ours” uses the MD-CLoss loss function. From the detection effect Figure 7, it can be seen that “Ours” can be well adapted to natural scene text detection and has higher accuracy.

## 5. Discussion

As the most widely used target detection algorithm in the industry, YOLOv3 has its irreplaceable detection advantages, but is limited by its output frame being a horizontal rectangle, so it cannot be adapted well to multi-directional scene text detection. To this end, this article uses the sliding vertex box definition method to apply it to the yolo layer of the YOLOv3 detection head, so that the algorithm has the function of multi-directional target positioning. For such changes, the MD-CLoss loss function and the step-by-step NMS method are used to ensure the efficiency of the algorithm.

We used comparison experiments with mainstream algorithms and new models in the past two years on the ICDAR2015 data set. In terms of detection accuracy, the algorithm in this paper proves that the algorithm has high detection accuracy with an accuracy rate of 86.2%. Among them, the MD-CLoss loss function makes a certain contribution to the accuracy improvement. Based on the CIOU, it effectively overcomes the problem of the IOU that cannot accurately reflect the position gap between the two frames. This paper used a comparative experiment to successfully prove the effectiveness of the MD-CLoss. In terms of time performance, compared with the high detection speed models EAST, Textboxes++, etc., the detection speed reached 21.3 fps, which shows a strong advantage.

The model in this paper has an accuracy of 86.2% on the ICDAR 2015 data set, which performs well. In particular, the model has a strong advantage in detection speed of 21.3 fps. This makes the model of this paper can be extended to the following scenarios: including real-time video detection and dynamic target tracking that require high model speed; or in practical applications where the equipment’s computing power is insufficient, but it is required to meet the basic detection speed.

## 6. Conclusions

This paper proposes a multi-directional scene text detection algorithm based on improved YOLOv3. On the basis of YOLOV3, the frame definition method of the sliding vertex is adopted to give the algorithm the function of multi-direction text detection. Then, a new rotating box loss function MD-Closs based on CIOU is proposed to improve the detection accuracy. In addition, a step-by-step NMS method is used to further reduce the amount of calculation. Experimental results show that the algorithm has an precision rate of 86.2% on the ICDAR 2015 data set, a recall rate of 81.9%, and a time performance of 21.3 fps. It has a good detection effect for text detection in natural scenes.

## Figures and Tables

**Table 1:** Comparison of improved algorithm and mainstream algorithm.

Methods	Precision	Recall	F
SegLink	73.1%	76.8%	75.0%
Hust_orientedText	77.3%	75.1%	76.2%
SSTD	79.3%	77.5%	78.1%
R2CNN	85.3%	80.7%	83.7%
PixeLink	85.2%	82.7%	83.9%
PAN	84.0%	81.9%	82.9%
DB(ResNet-50)	83.2%	91.8%	87.3%
Ours-I	83.6%	80.4%	82.3%
Ours	86.2%	81.9%	84.0%

**Table 2:** Runtime and performance comparison on ICDAR 2015 incidental text dataset.

Methods	Res	Device	FPS
EAST VGG16	720p	Titan X	13.2
EAST PVANET	720p	Titan X	16.8
Textboxes++	1024 × 1024	Titan X	11.6
PixeLink	720p	Titan X	7.3
TextFuseNet(ResNet-50)	1024 × 1024	Tesla V100	8.0
DB(ResNet-50)	640 × 640	GTX 1080Ti	12.0
ours	672 × 672	GTX 1080Ti	21.3

## Data Availability

Data sharing is not applicable to this article.

## References

[1] Li S., Cao W. (2021). SEMPANet: A modified path aggregation network with squeeze-excitation for scene text detection. Sensors.

[2] Voulodimos A., Doulamis N., Doulamis A., Protopapadakis E. (2018). Deep learning for computer vision: A brief review. Comput. Intell. Neurosci..

[3] Guo Y., Liu Y., Oerlemans A., Lao S., Wu S., Lew M.S. (2016). Deep learning for visual understanding: A review. Neurocomputing.

[4] Zhou P., Xu K., Wang D. (2018). Rail profile measurement based on line-structured light vision. IEEE Access.

[5] Redmon J., Divvala S., Girshick R., Farhadi A. You only look once: Unified, real-time object detection. Proceedings of the IEEE Conference on Computer Vision and Pattern Recognition.

[6] Ren S., He K., Girshick R., Sun J. (2016). Faster R-CNN: Towards real-time object detection with region proposal networks. IEEE Trans. Pattern Anal. Mach. Intell..

[7] Redmon J., Farhadi A. YOLO9000: Better, faster, stronger. Proceedings of the IEEE Conference on Computer Vision & Pattern Recognition.

[8] Redmon J., Farhadi A. (2018). Yolov3: An incremental improvement. arXiv.

[9] Liu W., Anguelov D., Erhan D., Szegedy C., Reed S., Fu C.-Y., Berg A.C. (2016). SSD: Single Shot Multibox Detector.

[10] Tian Z., Huang W., He T., He P., Qiao Y. (2016). Detecting Text in Natural Image with Connectionist Text Proposal Network.

[11] Shi B., Bai X., Belongie S. Detecting oriented text in natural images by linking segments. Proceedings of the IEEE Conference on Computer Vision and Pattern Recognition CVPR.

[12] Ye J., Chen Z., Liu J., Du B. TextFuseNet: Scene text detection with richer fused features. Proceedings of the Twenty-Ninth International Joint Conference on Artificial Intelligence and Seventeenth Pacific Rim International Conference on Artificial Intelligence.

[13] Xing L., Tian Z., Huang W., Scott M. Convolutional character networks. Proceedings of the 2019 IEEE/CVF International Conference on Computer Vision (ICCV).

[14] Liu Y., He T., Chen H., Wang X., Luo C., Zhang S., Shen C., Jin L. (2021). Exploring the capacity of an orderless box discretization network for multi-orientation scene text detection. Int. J. Comput. Vis..

[15] Ma J., Shao W., Ye H., Wang L., Wang H., Zheng Y., Xue X. (2018). Arbitrary-oriented scene text detection via rotation proposals. IEEE Trans. Multimed..

[16] Liao M., Shi B., Bai X. (2018). Textboxes++: A single-shot oriented scene text detector. IEEE Trans. Image Process..

[17] Xu Y., Fu M., Wang Q., Wang Y., Chen K., Xia G.-S., Bai X. (2020). Gliding vertex on the horizontal bounding box for multi-oriented object detection. IEEE Trans. Pattern Anal. Mach. Intell..

[18] He D., Xu K., Wang D. (2019). Design of multi-scale receptive field convolutional neural network for surface inspection of hot rolled steels. Image Vis. Comput..

[19] Zheng Z., Wang P., Ren D., Liu W., Ye R., Hu Q., Zuo W. (2020). Enhancing geometric factors in model learning and inference for object detection and instance segmentation. arXiv.

[20] Karatzas D., Gomez-Bigorda L., Nicolaou A., Ghosh S., Bagdanov A., Iwamura M., Matas J., Neumann L., Chandrasekhar V.R., Lu S. ICDAR 2015 competition on robust reading. Proceedings of the 2015 13th International Conference on Document Analysis and Recognition.

[21] Hu H., Zhang C.Q., Luo Y.X., Wang Y.Z., Han J., Ding E. Wordsup: Exploiting word annotations for character based text detection. Proceedings of the IEEE Conference on Computer Vision and Pattern Recognition CVPR.

[22] Jiang Y., Zhu X., Wang X., Yang S., Li W., Wang H., Fu P., Luo Z. R2CNN: Rotational Region CNN for Orientation Robust Scene Text Detection. Proceedings of the IEEE Conference on Computer Vision and Pattern Recognition CVPR.

[23] Dan D., Liu H., Li X., Cai D. (2018). PixelLink: Detecting Scene Text via Instance Segmentation. Proceedings of the IEEE Conference on Computer Vision and Pattern Recognition CVPR.

[24] Wang Z., Jiang J., Wu Y., Ye M., Bai X., Satoh S. (2019). Learning sparse and identity-preserved hidden attributes for person re-identification. IEEE Trans. Image Process..

[25] Liao M., Wan Z., Yao C., Chen K., Bai X. Real-time scene text detection with differentiable binarization. Proceedings of the Thirty-Fourth AAAI Conference on Artificial Intel-Ligence.

